# Integrating gene expression data via weighted multiple kernel ridge regression improved accuracy of genomic prediction

**DOI:** 10.1186/s12711-025-00997-9

**Published:** 2025-09-25

**Authors:** Xue Wang, Jingfang Si, Yachun Wang, Lingzhao Fang, Zhe Zhang, Yi Zhang

**Affiliations:** 1 State Key Laboratory of Animal Biotech Breeding, National Engineering Laboratory for Animal Breeding, Key Laboratory of Animal Genetics, Breeding and Reproduction of Ministry of Agriculture and Rural Affairs, College of Animal Science and Technology, China Agricultural University, Beijing, China; 2 The Center for Quantitative Genetics and Genomics (QGG), Aarhus University, 8000 Aarhus, Denmark; 3 Guangdong Provincial Key Lab of Agro-Animal Genomics and Molecular Breeding, College of Animal Science, South China Agricultural University, Guangzhou, China; 4 National Center of Technology Innovation for Dairy Industry, Hohhot, China

## Abstract

**Background:**

Gene expression profiles hold potentially valuable information for the prediction of breeding values and phenotypes. However, in practical breeding programs, most reference population individuals typically have only genomic data, lacking transcriptomic data. Predicting gene expression based on genetic markers and integrating the genetically predicted gene expression data into genomic prediction may offer a potential solution.

**Results:**

This study extends kernel ridge regression (KRR) to weighted multiple kernel ridge regression (WMKRR), which integrates genomic data and transcriptomic data predicted from genetic markers through a multiple kernel learning (MKL) approach. We evaluated the predictive ability of WMKRR compared to traditional genomic best linear unbiased prediction (GBLUP) and a combined genomic and transcriptomic best linear unbiased prediction (GTBLUP) in both genotype feature selection and non-feature selection scenarios in two datasets: (i) 3305 simulated data based on the Cattle Genotype-Tissue Expression (CattleGTEx) dataset, (ii) 5515 real dairy cattle data. Our results show that WMKRR yielded higher predictive abilities than GBLUP And GTBLUP in both simulated And real dairy cattle data. For the simulated data based on CattleGTEx, WMKRR achieved an average improvement in predictive ability of 1.12% And 1.13% over GBLUP And GTBLUP, respectively, under the non-feature selection scenario, And 3.17% And 3.23%, respectively, under the feature selection scenario. For the real dairy cattle data, in cross-validation, WMKRR improved over GBLUP And GTBLUP by An average of 5.56% And 7.23%, respectively, without feature selection, And by 5.66% And 6.40%, respectively, with feature selection. In forward validation, WMKRR improved over GBLUP And GTBLUP by An average of 5.68% And 8.41%, respectively, without feature selection, And by 4.66% And 7.06%, respectively, with feature selection.

**Conclusions:**

Our result demonstrates that the WMKRR model, which integrates genomic and genetically predicted transcriptomic data, achieves better prediction performance compared to traditional genomic prediction models. This study showed the potential of enhanced genomic breeding application using omics data with no further omics sequencing cost.

**Supplementary Information:**

The online version contains supplementary material available at 10.1186/s12711-025-00997-9.

## Background

Predicting complex traits is a fundamental objective of quantitative genetics. Since Meuwissen et al. [1] first proposed the term genomic selection (GS), it has been widely accepted and successfully implemented in animal [2] and plant [3] breeding programs. Schaeffer et al. [4] reported that the breeding costs using GS in dairy cattle could save up to 92% compared to traditional progeny testing. Statistical models are a key factor affecting the accuracy of genomic prediction. Currently, the most commonly used methods for genomic prediction include the genomic best linear unbiased prediction (GBLUP) [5], single-step GBLUP (ssGBLUP) [6, 7], least absolute shrinkage and selection operator (LASSO) [8], as well as the Bayesian methods such as BayesA, BayesB, and Bayes LASSO [9, 10] with various priors that use Markov chain Monte Carlo (MCMC) to estimate the required genetic parameters. Moreover, ssSNPBLUP [11], which is statistically equivalent to ssGBLUP, has been widely adopted in genetic evaluations of European dairy cattle, as it directly estimates marker effects and avoids creating and inverting the genomic relationship matrix. Liu et al [12]. presented formulas for predicting breeding values of genotyped selection candidates when solutions from ssSNPBLUP were available and the model had an residual polygenic (RPG) effect. Additionally, several other SNP augmented models have also been proposed to improve genomic prediction [13–16]. However, these linear models typically assume that genetic variants affect phenotypes only in an additive manner, failing to capture interactions between markers [17].

Machine learning (ML) techniques have received considerable attention in the field of genomic prediction in recent years and have been proposed for animal and plant breeding programs [18]. ML algorithms can flexibly and adaptively capture the hidden relationships between genotypes and phenotype by constructing complex nonlinear models, while making few or no specific distributional assumptions for predictors [19]. Kernel machine learning methods has proven to be a promising new tool capable of addressing the challenges presented by the explosive growth of genomic data [20]. Compared to traditional linear regression models, kernel machine learning methods exhibit some advantages, such as accommodation of high-dimensional genomic data, effective capture of nonlinear relationships, and flexible incorporation of structured information and computational complexity [20]. In recent years, kernel machine learning methods have increasingly been used in animal and plant genomic prediction [17, 21, 22]. As a kernel machine learning method, kernel ridge regression (KRR) applies a nonlinear kernel function in the original space to define an inner product in a high-dimensional transformed space, thereby offering generalization performance based on regularized least squares [21]. Recently, KRR has been demonstrated in multiple studies to provide effective predictions and high computational efficiency [17, 21, 23].

Advances in high-throughput sequencing technologies are providing a variety of multi-omics data complementary to genomics, such as gene expression, DNA methylation, and protein abundance, making it feasible to enhance prediction accuracy of complex traits. These data serve as bridges between genotypes and phenotypes, not only offering new dimensions for phenotype prediction but also providing connections between organismal phenotypes and genomic variations that are not easily captured at the genomic sequence level [24]. This has sparked interest in incorporating these data into genomic prediction models to improve prediction accuracy [25–27]. For example, transcriptomic quantification of gene expression can reflect the active portion of the genome and is readily accessible for use by plant and animal breeders [28]. Therefore, new strategies are needed to integrate these additional sources of information into genomic prediction models. Michel et al. [29] explored the integration of gene expression data into the prediction of wheat disease resistance traits by using a combined genomic and transcriptomic relationship matrix, showing that a hybrid matrix was superior to the genomic matrix. Morgante et al. [27] used a linear mixed model to incorporate genomic and transcriptomic information as random effects separately, and the results showed that integrating genomic and transcriptomic information improved the prediction accuracy for three quantitative traits in Drosophila. These benefits may be attributed to the additional genetic information that is implicitly captured by transcriptomic data, providing orthogonal insights from different biological perspectives for the prediction of complex traits. However, in practical breeding programs, due to the high cost of collecting multi-omics data, most reference individuals often possess only genomic data but lack transcriptomic data, which poses challenges for the large-scale integration of transcriptomic data into genomic prediction studies. Hu et al. [30] showed that some genes were genetically predictable genes, whose expressions can be accurately predicted with genetic markers. Accordingly, utilizing genetically predicted gene expression based on genotype inputs to assist genomic prediction may help improve prediction accuracy compared to using genotypes alone. More importantly, it can significantly reduce the need for omics measurements and associated costs. This is potentially advantageous for some tissues in dairy cattle that are difficult to sample in vivo (e.g., mammary, liver, heart, etc.). However, the effectiveness of using genetically predicted gene expression in genomic prediction has not been fully explored.

Multiple kernel learning (MKL) is an advanced technique in ML that combines multiple kernels to map data into another space (a process known as kernel fusion), and enhances predictive performance by optimizing the weights for each base kernel. Thereby, MKL not only learns the optimal data-dependent kernel for specific tasks but also constructs a sophisticated framework to integrate heterogeneous data sources for learning. These characteristics have led to widespread application of MKL in practice, including signal processing [31], bioinformatics data fusion [32] and object detection [33]. However, so far, few studies have utilized the MKL strategy to integrate omics data for predicting complex traits in animal and plant breeding. Therefore, given that the aforementioned advantages of KRR, extending it to a weighted multiple kernel format (weighted multiple kernel ridge regression (WMKRR)) by employing the MKL approach to integrate other omics data for prediction could be beneficial. In this study, we introduced an integrative strategy using WMKRR to combine genomic and genetically predicted transcriptomic data, and the effectiveness of this method was evaluated through both simulated data based on the Cattle Genotype-Tissue Expression (CattleGTEx) project and real dairy cattle data.

## Methods

### Ethics statement

The entire procedure for blood sample collection was carried out in strict accordance with the protocol approved by the Animal Care and Use Committee of China Agricultural University (permit number: AW42303202-2-1).

### Statistical models

#### GBLUP

The model of the GBLUP is given as

$${\mathbf{y}} = {\mathbf{1}}\upmu + {\mathbf{g}} + {\mathbf{e}},$$

where $\mathbf{y}$ is the vector of the response variable of genotyped individuals, that is, simulated phenotypes or de-regressed proofs (DRPs); $\upmu$ is the overall mean, and $\mathbf{1}$ is a vector of 1 s; $\mathbf{g}$ is the vector of genomic breeding values, following a distribution $N(\mathbf{0},\mathbf{G}{\sigma }_{\text{g}}^{2}$), where ${\sigma }_{\text{g}}^{2}$ is the additive genetic variance and $\mathbf{G}$ is the genomic relationship matrix (G matrix). The **G** matrix was calculated as $\frac{\mathbf{Z}{\mathbf{Z}}^{\prime}}{2\sum {p}_{i}(1-{p}_{i})}$ [5], in which ${p}_{i}$ represents the minor allele frequency (MAF) of marker $i$, and $\mathbf{Z}$ represents the MAF adjusted marker matrix with entries for genotypes AA and aa being (0–2 ${p}_{i}$) and (2–2 ${p}_{i}$), respectively. $\mathbf{e}\sim N(\mathbf{0},\mathbf{I}{\sigma }_{\text{e}}^{2}$) is the vector of random residuals, where $\mathbf{I}$ is the identity matrix and ${\sigma }_{\text{e}}^{2}$ is the residual variance.

#### GTBLUP

In GTBLUP (a combined genomic and transcriptomic best linear unbiased prediction, which differs from the ssGTBLUP [15] with T decomposition in the inverse of G matrix), gene expression data was integrated into genomic prediction. The GTBLUP model is given as follows:

$$\mathbf{y}=\mathbf{1}\upmu +\mathbf{g}+\mathbf{t}+\mathbf{e},$$

where $\mathbf{y}$, $\upmu$, $\mathbf{g}$, and $\mathbf{e}$ are as defined above. $\mathbf{t}$ is the vector of random individual transcriptomic effects with a normal distribution of $N(\mathbf{0},\mathbf{E}{\sigma }_{\text{t}}^{2}$). $\mathbf{E}= \frac{\mathbf{R}{\mathbf{R}}^{\mathbf{^{\prime}}}}{m}$ is the corresponding variance–covariance matrix, where, $\mathbf{R}$ is an $n\times m$ matrix of standardized gene expression levels for $n$ individuals and $m$ genes. The standardization of gene expression levels was conducted by calculating ${r}_{ij}=\frac{{x}_{ij}-{\overline{x} }_{j}}{{s}_{j}}$, where ${x}_{ij}$ is the expression level of gene $j$ in individual $i$, ${\overline{x} }_{j}$ is the average expression level of gene $j$ across all individuals, and ${s}_{j}$ is the standard deviation of gene $j$’s expression levels.

In this study, GBLUP and GTBLUP were carried out using DMU software [34]. The AI-REML method in the DMUAI procedure was used to estimate the variance components.

#### Weighted multiple kernel ridge regression (WMKRR)

Kernel ridge regression (KRR) is a nonlinear regression method that utilizes a nonlinear kernel function (${\varvec{\upphi}}({\mathbf{x}}_{\text{i}})$) to map the raw data into a high-dimensional feature space, and then constructs a ridge regression model within this feature space to make predictions [35]. This study extended KRR to WMKRR by applying a MKL strategy, allowing simultaneous incorporation of genotype data and gene expression data. The objective function of WMKRR can be expressed as:

$${\text{minL}}_{\text{WMKRR}}=\frac{1}{2}{\Vert {\varvec{\upbeta}}\Vert }^{2}+\frac{1}{2\uplambda }\sum_{\text{i}=1}^{\text{n}}{({\text{y}}_{\text{i}}-{{\varvec{\upbeta}}}^{\text{T}}{\varvec{\upphi}}({\mathbf{x}}_{\text{i}}))}^{2},$$

where $\uplambda$ is the regularization constant. The weight vector ${\varvec{\upbeta}}$ is determined by derivation as follows:

$${\varvec{\upbeta}}={({{\varvec{\upphi}}}^{\text{T}}{\varvec{\upphi}}+\uplambda \mathbf{I})}^{-1}{{\varvec{\upphi}}}^{\text{T}}\mathbf{y},$$

where ${\varvec{\upphi}}$ constitutes the mapped samples ${\varvec{\upphi}}({\mathbf{x}}_{\text{i}})$ in rows, and $\mathbf{I}$ denotes the identity matrix. ${\varvec{\upbeta}}$ can be formulated in terms of the dual weights *α* as follows:

$${\varvec{\upbeta}}=\sum_{\text{i}=1}^{\text{n}}{\alpha }_{\text{i}}{\varvec{\upphi}}\left({\mathbf{x}}_{\text{i}}\right)={{\varvec{\upphi}}}^{\text{T}}\alpha .$$

**Fig. 1:**
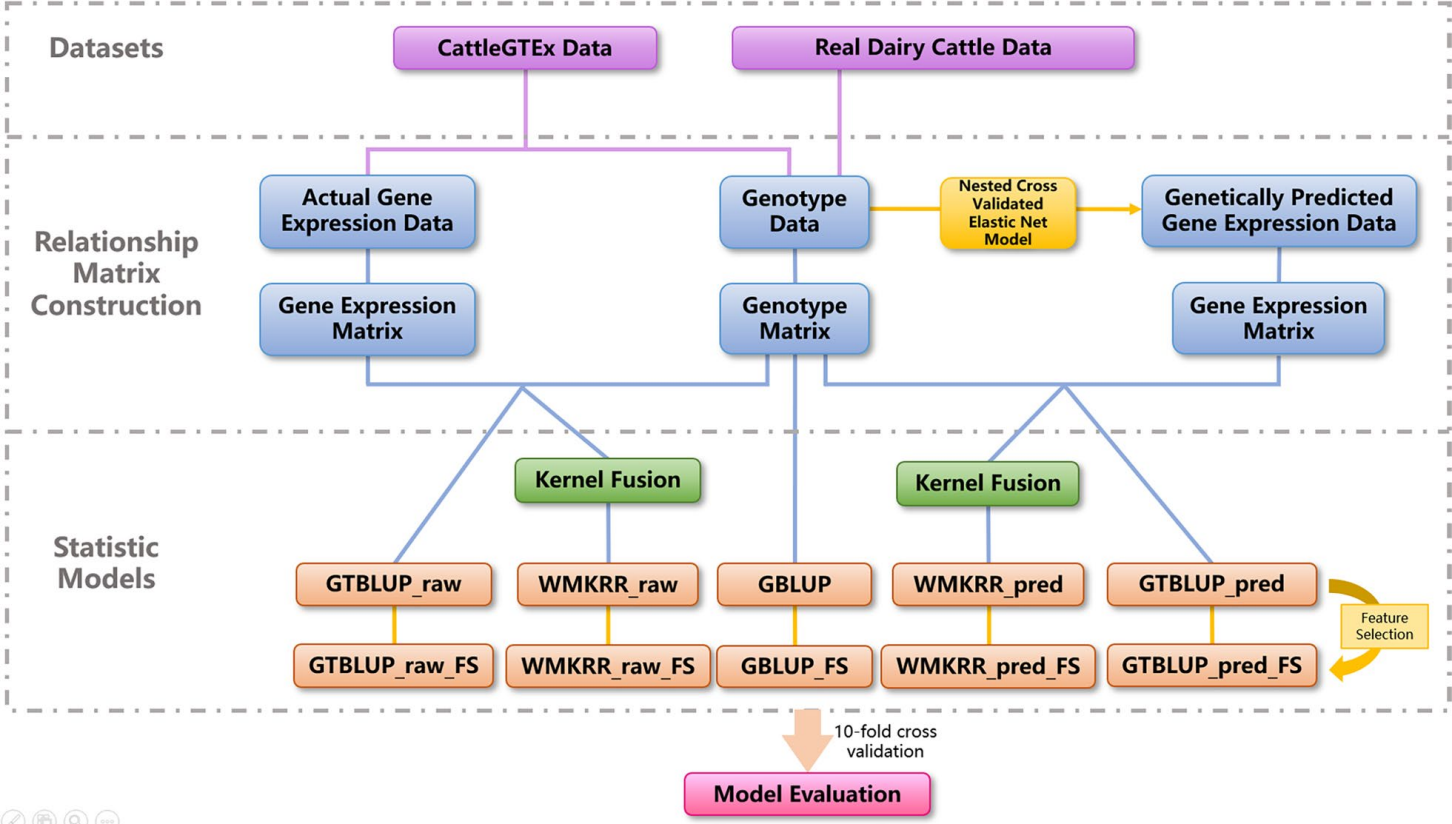
Flow diagram of experimental design

Therefore, the closed-form solution for the dual weight α is derived as follows:

$$\alpha ={({{\varvec{\upphi}}}^{\text{T}}{\varvec{\upphi}}+\uplambda \mathbf{I})}^{-1}\mathbf{y}={(\mathbf{K}+\uplambda \mathbf{I})}^{-1}\mathbf{y},$$

where $\mathbf{K}$ is the kernel matrix integrating genotype and gene expression data:

$$\mathbf{K}= {\text{w}}_{1}{\mathbf{K}}_{\mathbf{a}}+{\text{w}}_{2}{\mathbf{K}}_{\mathbf{b}}={\text{w}}_{1}\left[\begin{array}{ccc}\text{K}({\mathbf{x}}_{\mathbf{a}1},{\mathbf{x}}_{\mathbf{a}1})& \text{K}({\mathbf{x}}_{\mathbf{a}1},{\mathbf{x}}_{\mathbf{a}2})& \begin{array}{cc}\cdots & \text{K}({\mathbf{x}}_{\mathbf{a}1},{\mathbf{x}}_{\mathbf{a}\text{n}})\end{array}\\ \text{K}({\mathbf{x}}_{\mathbf{a}2},{\mathbf{x}}_{\mathbf{a}1})& \text{K}({\mathbf{x}}_{\mathbf{a}2},{\mathbf{x}}_{\mathbf{a}2})& \begin{array}{cc}\cdots & \text{K}({\mathbf{x}}_{\mathbf{a}2},{\mathbf{x}}_{\mathbf{a}\text{n}})\end{array}\\ \begin{array}{c}\vdots \\ \text{K}({\mathbf{x}}_{\mathbf{a}\text{n}},{\mathbf{x}}_{\mathbf{a}1})\end{array}& \begin{array}{c}\vdots \\ \text{K}({\mathbf{x}}_{\mathbf{a}\text{n}},{\mathbf{x}}_{\mathbf{a}2})\end{array}& \begin{array}{cc}\begin{array}{c}\vdots \\ \cdots \end{array}& \begin{array}{c}\vdots \\ \text{K}({\mathbf{x}}_{\mathbf{a}\text{n}},{\mathbf{x}}_{\mathbf{a}\text{n}})\end{array}\end{array}\end{array}\right]+{\text{w}}_{2}\left[\begin{array}{ccc}\text{K}({\mathbf{x}}_{\mathbf{b}1},{\mathbf{x}}_{\mathbf{b}1})& \text{K}({\mathbf{x}}_{\mathbf{b}1},{\mathbf{x}}_{\mathbf{b}2})& \begin{array}{cc}\cdots & \text{K}({\mathbf{x}}_{\mathbf{b}1},{\mathbf{x}}_{\mathbf{b}\text{n}})\end{array}\\ \text{K}({\mathbf{x}}_{\mathbf{b}2},{\mathbf{x}}_{\mathbf{b}1})& \text{K}({\mathbf{x}}_{\mathbf{b}2},{\mathbf{x}}_{\mathbf{b}2})& \begin{array}{cc}\cdots & \text{K}({\mathbf{x}}_{\mathbf{b}2},{\mathbf{x}}_{\mathbf{b}\text{n}})\end{array}\\ \begin{array}{c}\vdots \\ \text{K}({\mathbf{x}}_{\mathbf{b}\text{n}},{\mathbf{x}}_{\mathbf{b}1})\end{array}& \begin{array}{c}\vdots \\ \text{K}({\mathbf{x}}_{\mathbf{b}\text{n}},{\mathbf{x}}_{\mathbf{b}2})\end{array}& \begin{array}{cc}\begin{array}{c}\vdots \\ \cdots \end{array}& \begin{array}{c}\vdots \\ \text{K}({\mathbf{x}}_{\mathbf{b}\text{n}},{\mathbf{x}}_{\mathbf{b}\text{n}})\end{array}\end{array}\end{array}\right],$$

where ${\text{w}}_{1}+{\text{w}}_{2}=1$, ${\mathbf{K}}_{\mathbf{a}}$ and ${\mathbf{K}}_{\mathbf{b}}$ are the kernel matrices (linear kernel or Gaussian kernel) constructed from genotype and gene expression data, respectively, and $\text{K}\left({\mathbf{x}}_{\mathbf{a}\text{i}},{\mathbf{x}}_{\mathbf{a}\text{j}}\right)={\varvec{\upphi}}({\mathbf{x}}_{\mathbf{a}\text{i}})\cdot {{\varvec{\upphi}}({\mathbf{x}}_{\mathbf{a}\text{j}})}^{\text{T}}$ and $\text{K}\left({\mathbf{x}}_{\mathbf{b}\text{i}},{\mathbf{x}}_{\mathbf{b}\text{j}}\right)={\varvec{\upphi}}({\mathbf{x}}_{\mathbf{b}\text{i}})\cdot {{\varvec{\upphi}}({\mathbf{x}}_{\mathbf{b}\text{j}})}^{\text{T}}$. In a Gaussian kernel, the distance between individuals $(\text{i},\text{j})$ is represented as $\text{K}\left({\mathbf{x}}_{\text{i}},{\mathbf{x}}_{\text{j}}\right)=\prod_{\text{k}=1}^{\text{p}}\text{exp}(-\theta {({\text{x}}_{\text{ik}}-{\text{x}}_{\text{jk}})}^{2})$, where $\theta$ is a positive bandwidth parameter and ${\text{x}}_{\text{ik}}(\text{i},\text{j}=1,\dots ,\text{n},\text{k}=1,\dots ,\text{p})$ is the SNP genotype (or gene expression) for individual $\text{i}$ at SNP (or gene) $\text{k}$. Finally, for a new test sample ${\mathbf{x}}_{\text{i}}$, the predicted output is derived using dual weights and the similarity between the test sample ${\mathbf{x}}_{\text{i}}$ and all training samples used in the prediction. Hence, the expression for WMKRR is:

$$\text{y}\left({\mathbf{x}}_{\text{i}}\right)={\mathbf{k}}^{\prime}{{\left(\mathbf{K}+\uplambda \mathbf{I}\right)}^{-1}}\mathbf{y},$$

where ${\mathbf{k}}^{\boldsymbol{^{\prime}}}={\text{w}}_{1}\mathbf{K}({\mathbf{x}}_{\mathbf{a}\text{i}},{\mathbf{x}}_{\mathbf{a}\text{j}})+{\text{w}}_{2}\mathbf{K}({\mathbf{x}}_{\mathbf{b}\text{i}},{\mathbf{x}}_{\mathbf{b}\text{j}})$ (j = 1, 2, 3, …, n). Notably, the optimal kernel weights ${\text{w}}_{1}$, ${\text{w}}_{2}$, regularization constant $\uplambda$, and the Gaussian kernel bandwidth parameter $\theta$ was automatically determined by using Bayesian optimization algorithm [36]. Bayesian optimization is a global optimization method based on Gaussian process regression, efficiently finding optimal parameters by balancing exploration and exploitation. The process of Bayesian optimization is as follows: (i) Initial evaluation: Randomly select several hyperparameter combinations (i.e., ${\text{w}}_{1}$, ${\text{w}}_{2}$, $\uplambda$ and $\theta$ in this study), evaluate their performance based on cross-validation, and construct a Gaussian process as a surrogate model. (ii) Posterior update: Update the posterior distribution of the Gaussian process based on the initial evaluation results. (iii) Acquisition function optimization: Use the acquisition function (i.e., expected improvement) to determine the next hyperparameter combination to be evaluated. (iv) Iterative update: Repeat steps 2 And 3, continuously evaluate new hyperparameter combinations and update the surrogate model until the set number of iterations is reached (200 iterations in this study). (v) Optimal parameter selection: After completing all iterations, select the hyperparameter combination that performs best (using Pearson correlation in this study) across all evaluations as the final optimal hyperparameters. In this study, the Bayesian optimization algorithm was implemented based on scikit-learn library [37]. The WMKRR software is available on GitHub at https://github.com/Wangxuer521/WMKRR/tree/master. The experimental design of this study is illustrated in Fig. 1.

### Data simulation

#### Data sets

Whole-genome sequence (WGS) data containing 48,811,382 imputed SNPs And gene expression profiles consisting of 27,607 genes extracted from transcriptome data were collected from the Cattle Genotype-Tissue Expression (CattleGTEx) project [38]. A total of 3305 individuals from 24 purebred And 11 crossbred with both imputed WGS data And gene expression data were selected for Analysis, of which 1210, 774, 721 And 600 individuals had gene expression data for mammary, muscle, blood, and Liver tissues, respectively. For each tissue, SNPs with a MAF lower than 0.01 were excluded using PLINK software [39]. A total of 19,073,630, 16,006,668, 9,213,817 And 7,888,127 SNPs were retained for further analysis in muscle, liver, blood, and mammary tissue, respectively. For the gene expression data, the raw format was transcripts per million (TPM), and a ${\text{log}}_{2}(\text{TPM}+1)$ transformation was performed for further analysis. In addition, due to the lack of phenotype data in the CattleGTEx project, a phenotypic simulation was performed, with the simulation strategy described below.

#### Gene expression prediction

To compare the genomic prediction performance between integrating genetically predicted gene expression and integrating actual gene expression, we not only used the actual gene expression values from the CattleGTEx data, but also predicted the expression values of genetically predictable genes in each tissue based on genotype data using the Nested Cross Validated Elastic Net prediction models constructed by Liu et al. [38]. We first retrieved the SNPs required for predicting gene expression in each tissue from the prediction model database and subsequently extracted these SNPs from the WGS data for gene expression prediction. The number of SNPs for gene expression prediction in muscle, Liver, blood, And mammary tissues were 117,803, 120,007, 215,898 And 48,747, respectively, with the corresponding number of genetically predictable genes being 2621, 3112, 4750 And 1898, respectively. Additionally, these SNPs and genes were also used for subsequent genomic prediction analyses. Gene expression prediction accuracy was measured by Spearman correlation coefficient between the predicted and the observed gene expression values.

#### Cis-heritability estimation of gene expression

The accuracy of gene expression prediction is affected by the cis-heritability of gene expression [30]. To understand the overall contribution of cis-genetic variants to variation in gene expression level, restricted maximum likelihood method (REML) was employed to estimate the cis-heritability of each gene based on the gene expression And cis-SNPs located within 1 Mb up- and down-stream of the physical position of the gene [40]. For the estimation of cis-heritability of gene expression in each tissue, we adjusted gene expression for hidden confounding factors and underlying population structure using Probabilistic Estimation of Expression Residuals (PEER) factors and genotype principal components (PCs), with the first five genotype PCs and ten PEER factors included as covariates in the model [40]. PEER factors for each tissue were estimated using the PEER method implemented in the PEER R package [41], and principal component analysis (PCA) was performed for each tissue using GCTA software [42], where the first five PCs explained 27.13%, 23.95%, 6.54%, And 37.96% of the total variance in muscle, liver, blood, and mammary tissues, respectively.

#### Phenotype simulation

After estimating the cis-heritability of gene expression, SNPs corresponding to genes with gene expression cis-heritability greater than 0.8 And gene expression prediction accuracy greater than 0.5 were selected from the previously mentioned genes and SNPs used for genomic prediction (with 4616, 2797, 1734, And 1351 SNPs for blood, liver, mammary and muscle tissues, respectively). Subsequently, quantitative trait loci (QTL) were generated through simple random sampling without replacement from these SNPs. In this study, the genetic structure of quantitative traits is considered based on heritability and the number of QTLs, with the assumption that the genotype influences the phenotype in a linear manner. Following Ren et al. [43], heritability And number of QTLs were set to 0.8 And 100, respectively, and the phenotypic variance ${\sigma }_{p}^{2}$ was set to 1. The allele substitution effect of the ${i}^{th}$ QTL, ${a}_{i}$, was calculated using ${a}_{i}={(2{p}_{i}(1-{p}_{i}))}^{-1/2}{\sigma }_{g}/\sqrt{m}$, where ${\sigma }_{g}=\sqrt{{\sigma }_{p}^{2}\times {h}^{2}}$ denotes the genetic standard deviation attributed to all QTLs, ${p}_{i}$ represents the frequency of a specific allele for the ${i}^{th}$ QTL and $m$ is the total count of QTLs. The residual effects followed a normal distribution $N(0, (1-{h}^{2}){\sigma }_{p}^{2})$. The genetic values (i.e., true breeding values (TBVs)) for each individual were calculated by combing the individual’s genotype information with the QTL effects. The simulated phenotype value of each individual was calculated as the sum of TBV And residual effects, with each simulation replicated 10 times for each tissue. The code for phenotype simulation is available on GitHub at https://github.com/Wangxuer521/Phe_simulation_code/tree/master.

### Real dairy cattle data

#### Data sets

**Fig. 2:**
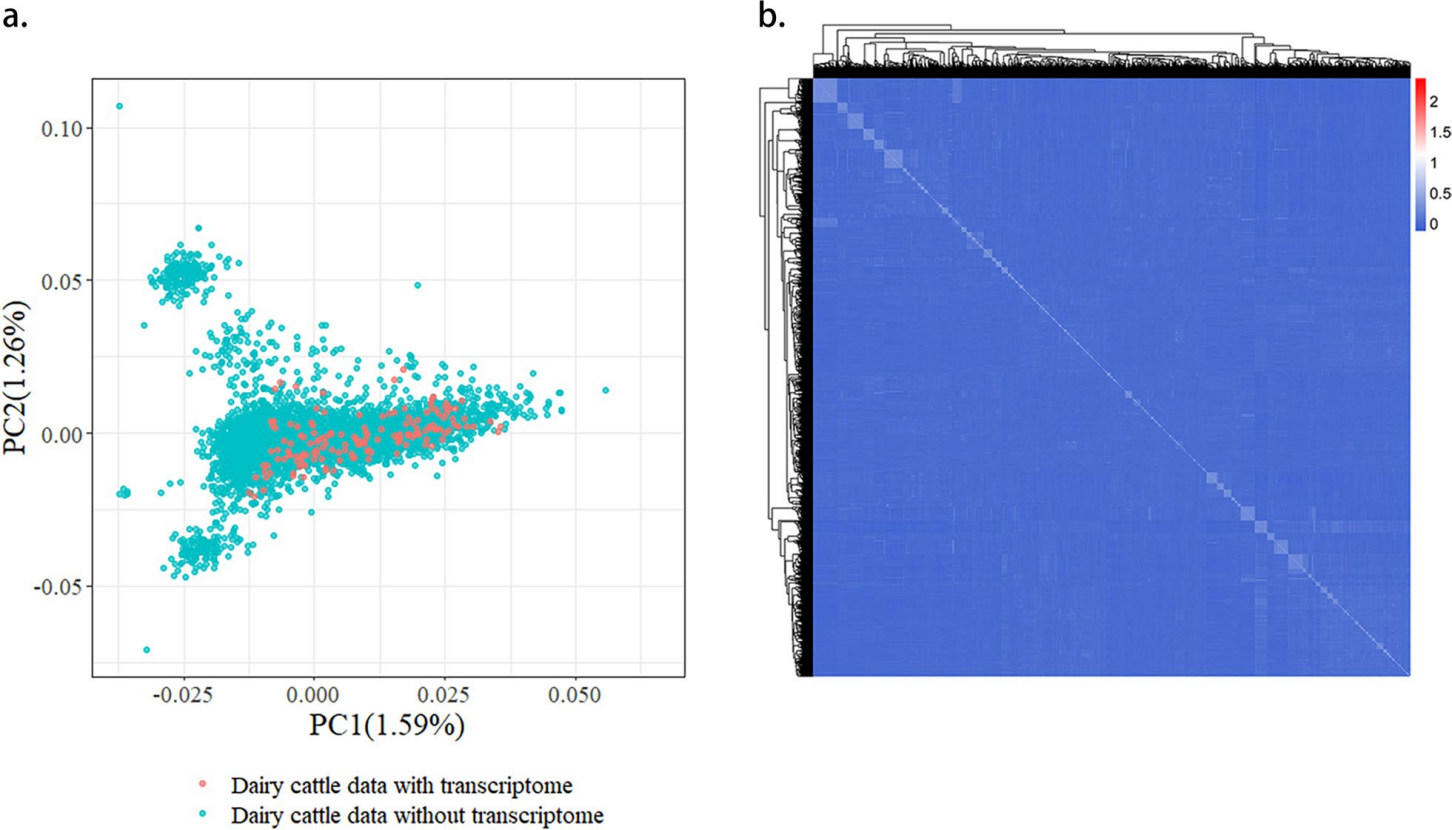
Principal component analysis and genomic relationship matrix heat map of the real dairy cattle data. **a** Principal component analysis. **b** Genomic relationship matrix heat map. PC1: first principal component, PC2: second principal component

To explore the genomic prediction performance of WMKRR in real dairy cattle data, phenotypic And genomic data were collected from 5515 Chinese Holstein cattle. All animals were genotyped using the BovineSNP50 chip containing 54,609 SNPs from Illumina (Illumina, San Diego, CA, USA). Imputation of missing genotypes was carried out using Beagle 5.4 [44]. After imputation, SNPs with MAF lower than 0.01 and significantly deviating from Hardy–Weinberg equilibrium (*P* < 1.0E-6) were removed using PLINK software [39]. After quality control, 45,254 autosomal SNPs were retained for subsequent genomic prediction. The phenotypic data included three milk production traits: milk yield (MY), fat yield (FY), and protein yield (PY). The method of Jairath et al. [45] was used to derive DRPs from official estimated breeding values (EBV) provided by the Dairy Association of China. These were utilized as pseudo-phenotypes for genomic prediction. The DRP reliability of each individual was estimated as ${r}_{DRP}^{2}=\frac{{ERC}_{i}}{{ERC}_{i}+\lambda }$, with $\lambda =\frac{1-{h}^{2}}{{h}^{2}}$, where ${ERC}_{i}$ denotes the effective record contribution and ${h}^{2}$ denotes the heritability of the trait (0.3, 0.25 And 0.28 for MY, FY and PY traits, respectively). Notably, ${ERC}_{i}=\lambda *\frac{{REL}_{i}}{1-{REL}_{i}}$, where ${REL}_{i}$ represents the reliability of EBV for individual $i$. Among the 5515 dairy cattle, we collected blood samples from 157 dairy cows in the same lactation period from the same farm to generate transcriptomic data. Transcriptome sequencing (RNA-seq) for these cows was performed on the Illumina NovaSeq 6000 platform, generating paired-end reads of 2 × 150 bp, with a total of 27,421 genes quantified. Figure 2a shows the PCA of the 157 cows with transcriptome data And the remaining 5358 individuals without transcriptome data, and Fig. 2b illustrates the heat map of genomic relationship matrix for all 5515 dairy cattle. The pedigree relationship between the 157 cattle And the remaining 5358 cattle is shown in Table S1 [see Additional file 1: Table S1].

#### Gene expression prediction

For the 5515 real dairy cattle, we used the Nested Cross Validated Elastic Net prediction model [38] to predict the gene expression values in blood tissue based on the genotypic data of all individuals. Since the gene expression prediction model requires a significantly larger number of SNPs than those included in the 50 K SNP panel, the BovineSNP50 chip was imputed to WGS level using Beagle 5.4 [44], with the reference population consisting of 1842 cattle from the 1000 Bull Genomes Project [46]. Variants with a MAF lower than 0.01 were excluded using the PLINK software [39], resulting in 34,859,114 SNPs from the imputed WGS data. We then selected 199,650 SNPs required for gene expression prediction And selected the corresponding 4739 genetically predictable genes for subsequent analyses. The theoretical imputation accuracy was assessed by the dosage R-squared (DR^2^), which is the estimated squared correlation between the estimated allele dose And the true allele dose calculated using Beagle 5.4 [44]. In addition, gene expression prediction accuracy was measured by Spearman correlation coefficient between the predicted And the observed gene expression values for the 157 cattle with transcriptome data.

#### Cis-heritability estimation of gene expression

Since the accuracy of gene expression prediction is influenced by its cis-heritability [30], we assessed the effectiveness of gene expression prediction in real dairy cattle data by estimating the cis-heritability of each gene’s expression using the actual transcriptomic data from 157 dairy cows And calculating the correlation coefficient between the cis-heritabilities of these gene expressions And their prediction accuracies. The estimation method for cis-heritability here was the same as that used in the analysis of the CattleGTEx data. It should be noted that the genotype data used here was the WGS level data after imputation using Beagle 5.4 [44] and quality control (MAF > 0.01) using PLINK software [39], with a total of 9,186,844 SNPs retained.

### Cross-validation and genomic predictive ability

In this study, a 5 × 10 cross-validation (five repeats of ten-fold CV) was performed to assess the predictive ability, root mean square error (RMSE) and unbiasedness of different methods. Notably, the reference and validation populations remained constant for all methods in each replicate of ten-fold CV, and the final prediction results were the averages of five replicates. The predictive ability was assessed for the CattleGTEx data as the Pearson correlation between simulated phenotypes and predicted values (PV) of the validation population. For the real dairy cattle data, the predictive ability was further corrected by the mean accuracy $\overline{r }$ (square root of reliability) of DRP in validation population:

$$Predictive\space ability=\frac{cor\left(DRP, PV\right)}{\overline{r} }.$$

In addition, prediction unbiasedness was calculated as the regression of simulated phenotypes (in the CattleGTEx data) or DRP (in the real dairy cattle data) on PV of the validation individuals. Moreover, the formula of RMSE can be written as follows:

$$RMSE=\sqrt{\frac{\sum_{i=1}^{n}{({PV}_{i}^{\prime}-{y}_{i}^{\prime})}^{2}}{n},}$$

where $n$ represents the number of individuals in the validation population, ${PV}_{i}$ represents the centralized predicted values, and ${y}_{i}$ represents the centralized observed values (simulated phenotypes or DRPs).

Additionally, for the real dairy cattle data, we also compared the performance of each method in a forward validation scenario (i.e., using early-generation animals to predict the performance of later-generation animals). The birth years ranged from 1993 to 2019, with the majority (96%) of the cattle born between 2003 And 2019. Therefore, the youngest cattle born in the 2017–2019 period (842 cattle) were selected as the validation population, while the remaining 4,673 cattle were used as the reference population. Predictive ability, unbiasedness and RMSE were calculated as described above.

### Model comparison

As shown in Fig. 1, for the CattleGTEx data, the GTBLUP method based on genetically predicted and actual gene expression data are denoted as GTBLUP_pred and GTBLUP_raw, respectively; similarly, the WMKRR methods based on genetically predicted and actual gene expression data are referred to as WMKRR_pred and WMKRR_raw, respectively. For the real dairy cattle data, in addition to using genotype data, the GTBLUP and WMKRR methods based on genetically predicted gene expression values are denoted as GTBLUP_pred and WMKRR_pred, respectively. For all prediction methods, in addition to utilizing all SNPs, univariate feature selection [47] was also performed on the genotype data to select the top 30,000 SNPs for the construction of the **G** matrix or the ${\mathbf{K}}_{\mathbf{a}}$ matrix and the evaluation of the predictive performance. It should be noted that for each CV fold, feature selection was applied separately to ensure that the marker selection was not based on any information from the validation set. The methods with feature selection were termed as GBLUP_FS, GTBLUP_pred_FS, GTBLUP_raw_FS, WMKRR_pred_FS, and WMKRR_raw_FS, respectively. Since the phenotypes simulated in the CattleGTEx data have a linear relationship with genotypes (see above for details), the use of nonlinear kernels is not expected to capture nonlinearity. Therefore, linear kernels were used for the kernel matrices in WMKRR for the CattleGTEx data. However, the real dairy cattle data sets contained actual phenotypes, which often exhibit complex nonlinear relationships with genotypes and gene expression. Therefore, nonlinear Gaussian kernels were used in WMKRR for the real dairy cattle data to capture the nonlinear structures.

## Results

### CattleGTEx data with simulated phenotypes

#### Correlation between gene expression cis-heritability and gene expression prediction accuracy

**Fig. 3:**
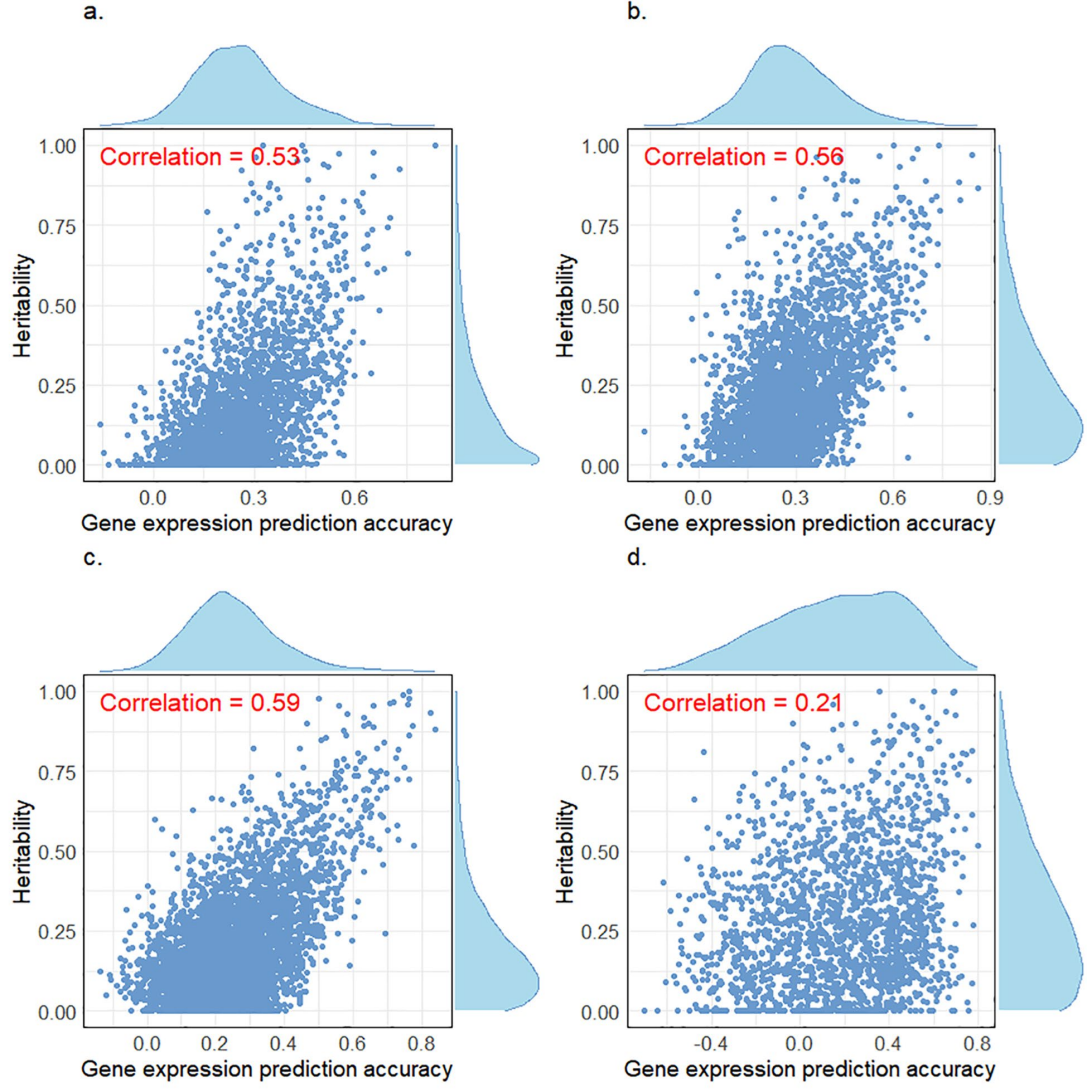
Correlation between gene expression heritability and gene expression prediction accuracy in various tissues, using the CattleGTEx data. **a** Muscle. **b** Liver. **c** Blood. **d** Mammary

The average DR^2^ value for the SNPs used in gene expression prediction was 0.84. Figure S1 [see Additional file 2: Figure S1] illustrates the DR^2^ values across different MAF intervals and for each chromosome. Figure 3 illustrates the correlation between gene expression cis-heritability and gene expression prediction accuracy, along with their respective distributions. The average gene expression prediction accuracy for all predicted genes in muscle, Liver, blood And mammary tissues were 0.26, 0.29, 0.25, And 0.17, with standard deviations of 0.13, 0.14, 0.13, And 0.30, respectively. Pearson correlation coefficients between gene expression cis-heritability And gene expression prediction accuracy for each genetically predictable gene were 0.53, 0.56, 0.59 And 0.21 for muscle, liver, blood and mammary tissues respectively. These results indicate a strong linear relationship between gene expression prediction accuracies and gene expression cis-heritabilities.

#### Genomic predictive ability

##### Model performance without feature selection

**Fig. 4:**
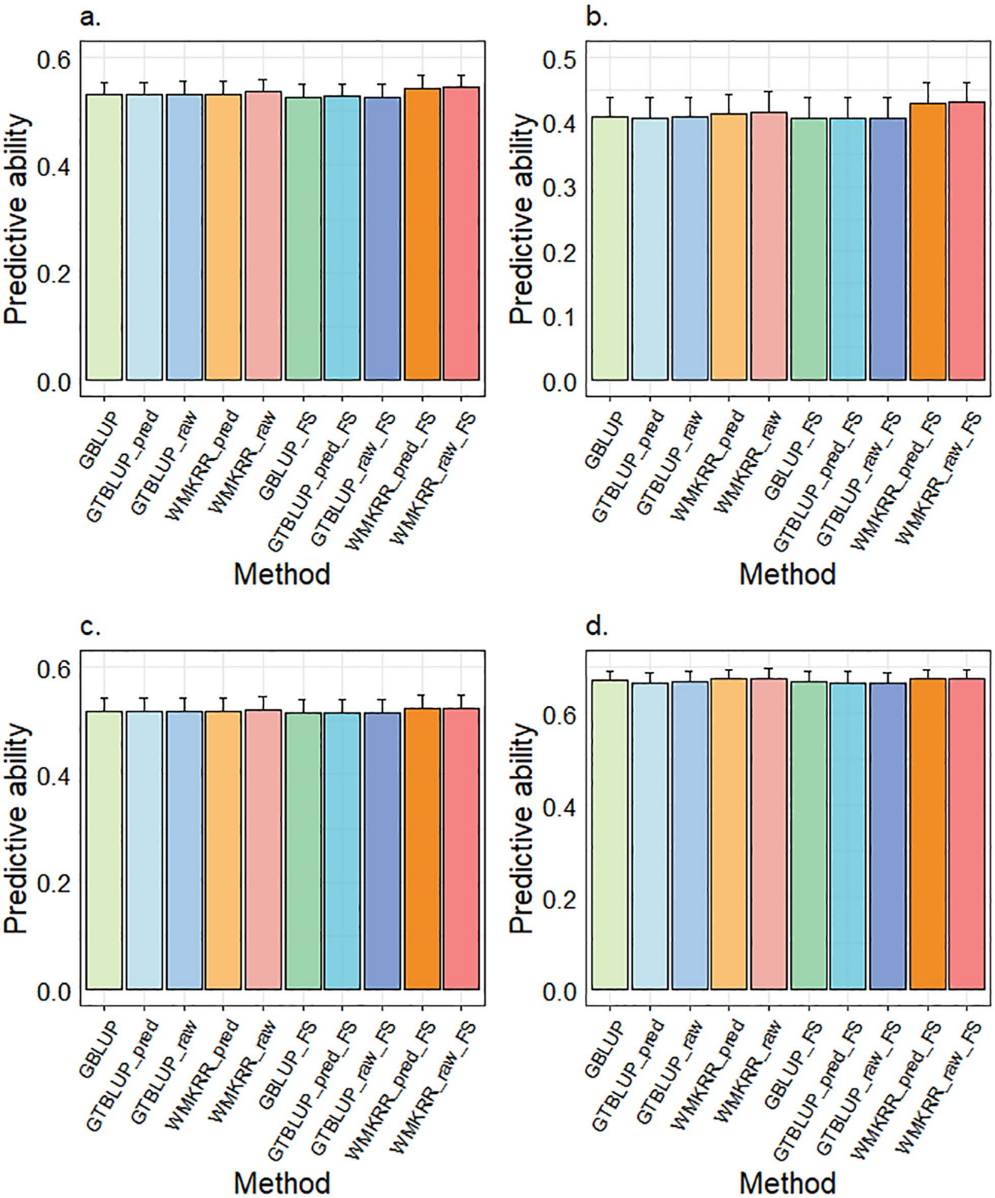
Predictive ability of genomic prediction based on the CattleGTEx data. **a** Muscle. **b** Liver. **c** Blood. **d** Mammary. GTBLUP_pred/raw: the GTBLUP method based on genotype and predicted/actual gene expression data; WMKRR_pred/raw: the WMKRR method based on genotype and predicted/actual gene expression data; GBLUP_FS: GBLUP with feature selection; GTBLUP_pred/raw_FS: GTBLUP_pred/raw with feature selection; WMKRR_pred/raw_FS: WMKRR_pred/raw with feature selection

Figure 4 presents the average predictive ability of genomic prediction for the CattleGTEx data (the raw values shown in Table S2 [see Additional file 1: Table S2]). In absence of feature selection, WMKRR_raw achieved the highest predictive ability across all tissues, but the average improvement in predictive ability of WMKRR_raw over traditional GBLUP, GTBLUP_pred, and GTBLUP_raw across all tissues were only 1.12%, 1.43%, And 1.13%, respectively. In addition, although WMKRR_pred achieved suboptimal overall predictive ability, it only achieved An average improvement of 0.52%, 0.84%, And 0.54% in predictive ability compared to traditional GBLUP, GTBLUP_pred, and GTBLUP_raw, respectively. Moreover, it was found that the predictive ability based on genetically predicted gene expression was nearly equivalent to that based on actual gene expression. Specifically, GTBLUP_raw and WMKRR_raw only showed An average improvement of 0.29% And 0.59% respectively over GTBLUP_pred and WMKRR_pred across all tissues. Moreover, it was observed that the predictive abilities of both GTBLUP_pred and GTBLUP_raw were close to that of GBLUP. In all scenarios WMKRR_raw achieved the lowest RMSE values compared to other methods, while no obvious differences in bias were observed among different methods, as the slopes of regression were close to 1 for all methods [see Additional file 1: Table S2].

##### Impact of feature selection on predictive ability

When feature selection was performed, higher predictive ability was obtained using WMKRR over conventional GBLUP and GTBLUP methods, as shown in Fig. 4. Specifically, WMKRR_pred_FS showed average improvements of 2.75%, 2.82%, And 2.82% over GBLUP_FS, GTBLUP_pred_FS, and GTBLUP_raw_FS across all tissues, ranging from 0.67 to 5.55%, 1.13 to 5.55%, And 1.27 to 5.55%, respectively. The predictive ability of WMKRR_raw_FS improved by An average 3.17%, 3.23%, And 3.23% over GBLUP_FS, GTBLUP_pred_FS, and GTBLUP_raw_FS, respectively across all tissues, ranging from 0.82 to 6.29%, 1.28 to 6.29%, And 1.42 to 6.29%. In addition, it was observed that genomic prediction performance based on genetically predicted gene expression was comparable to that based on actual gene expression, i.e., GTBLUP_pred_FS and WMKRR_pred_FS respectively produced predictive abilities similar to those of GTBLUP_raw_FS and WMKRR_raw_FS. Moreover, after feature selection on genotype data, the predictive ability of WMKRR improved, while no improvement was observed in the predictive abilities of GBLUP and GTBLUP. Specifically, WMKRR_pred_FS and WMKRR_raw_FS showed An average improvement of 1.68% And 1.49%, respectively over WMKRR_pred and WMKRR_raw across all tissues, while GBLUP_FS, GTBLUP_pred_FS, and GTBLUP_raw_FS showed An average decrease of 0.52%, 0.27%, And 0.56% over GBLUP, GTBLUP_pred, and GTBLUP_raw, respectively. As shown in Table S2 [see Additional file 1: Table S2], WMKRR_raw_FS achieved the lowest RMSE in all scenarios, and WMKRR_pred_FS exhibited the second lowest RMSE. No obvious differences in bias were observed among the different methods.

### Real dairy cattle data

#### Correlation between gene expression cis-heritability and gene expression prediction accuracy

**Fig. 5:**
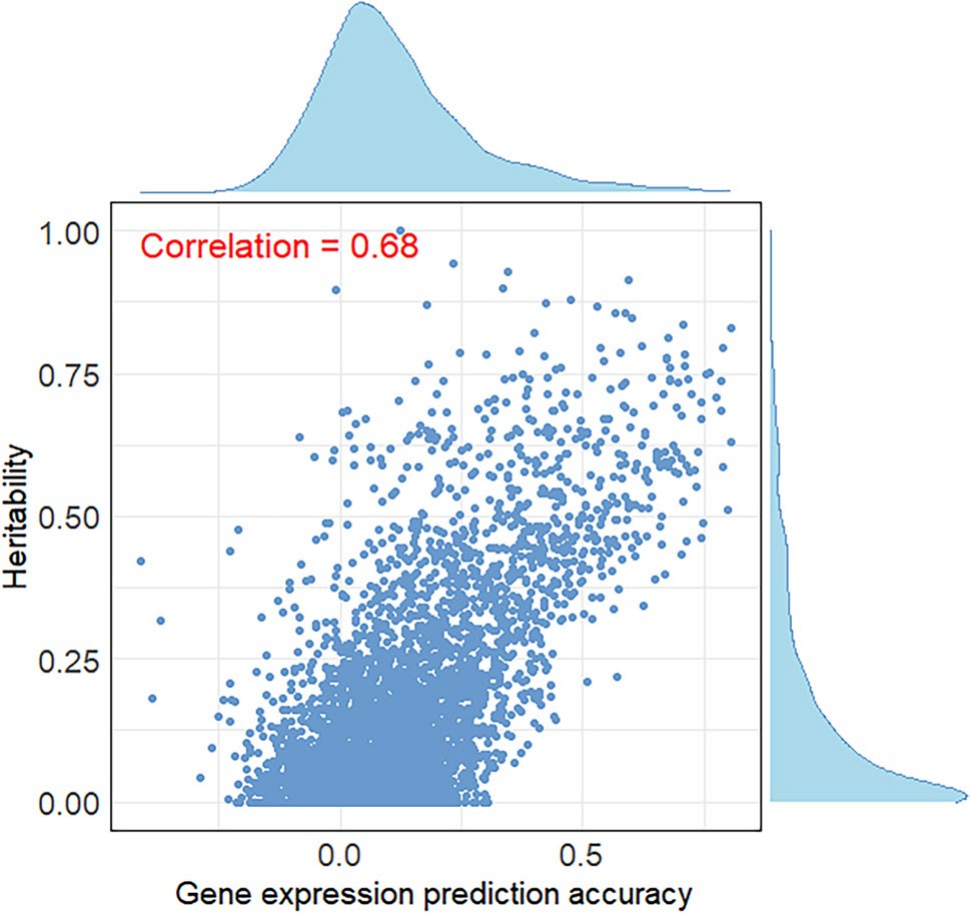
Correlation between gene expression heritability and gene expression prediction accuracy in the real dairy cattle data with transcriptome

Since actual transcriptomic data is available for 157 cattle in the real dairy data, we evaluated the effectiveness of gene expression prediction for these cattle. Figure 5 shows the Pearson correlation coefficient between gene expression cis-heritability And gene expression prediction accuracy, along with their respective distributions. The average gene expression prediction accuracy for all predicted genes was 0.12, with a standard deviation of 0.16. In addition, the Pearson correlation coefficient between gene expression cis-heritability And gene expression prediction accuracy was 0.68, indicating a strong linear relationship between them.

#### Genomic predictive ability in cross-validation scenario

**Fig. 6:**
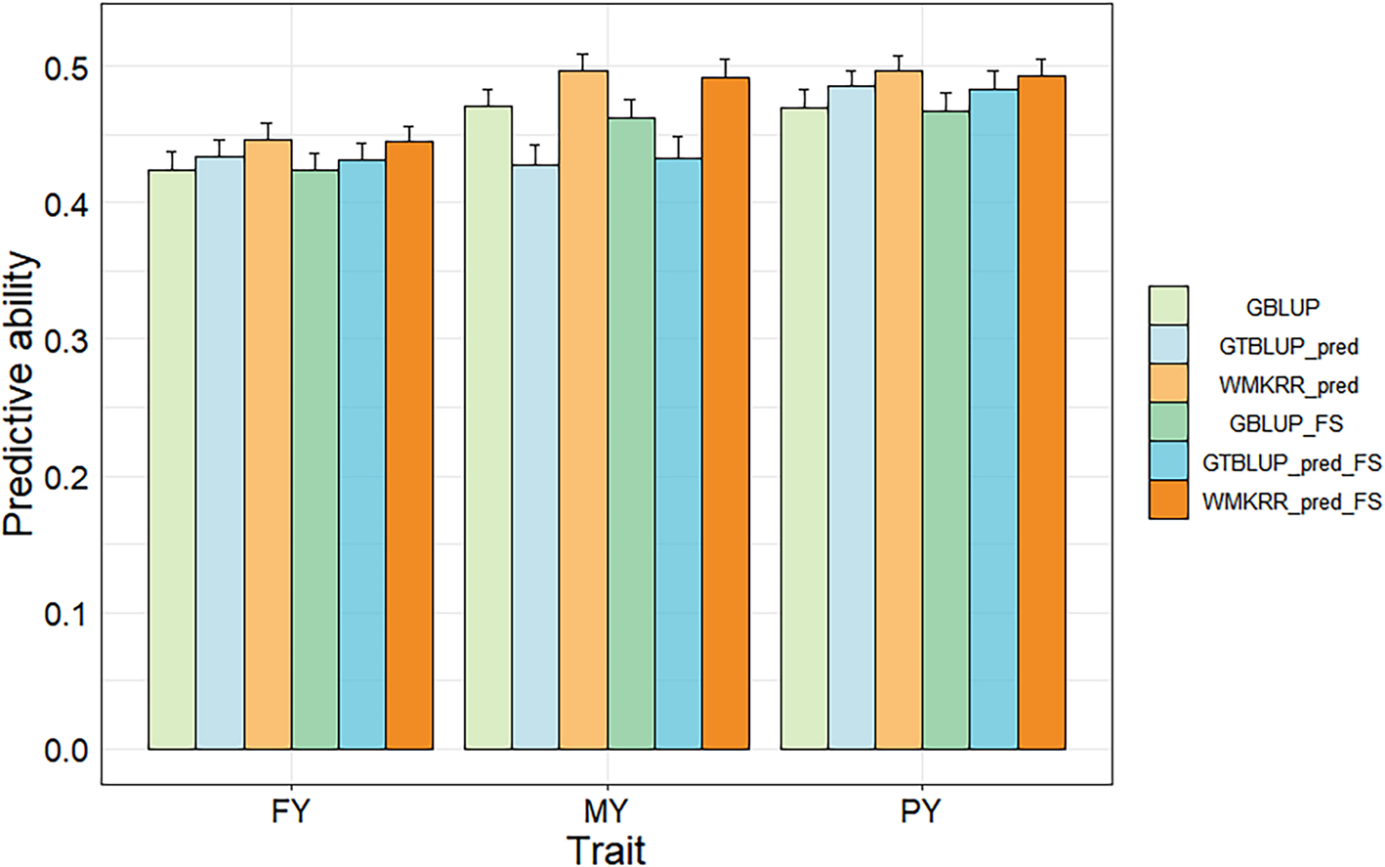
Predictive ability of genomic prediction on the real dairy cattle data in cross-validation. FY: fat yield; MY: milk yield; PY: protein yield. GTBLUP_pred: the GTBLUP method based on genotype and predicted gene expression data; WMKRR_pred: the WMKRR method based on genotype and predicted gene expression data; GBLUP_FS: GBLUP with feature selection; GTBLUP_pred_FS: GTBLUP_pred with feature selection; WMKRR_pred_FS: WMKRR_pred with feature selection

The genomic predictive abilities for three milk production traits on the real dairy cattle data in cross-validation are shown in Fig. 6. Although no actual gene expression data was available, the WMKRR method based on the genetically predicted gene expression (i.e., WMKRR_pred) still demonstrated higher predictive ability. Without feature selection, WMKRR_pred achieved An average improvement of 5.56% And 7.23% across all traits compared to traditional GBLUP and GTBLUP_pred, ranging from 5.19 to 5.76% And 2.27 to 16.67%, respectively. When comparing GBLUP with GTBLUP, it was found that the predictive ability of GTBLUP_pred based on genetically predicted gene expression was superior to that of GBLUP for FY and PY traits (improved by 2.36% And 3.41%, respectively), but inferior to that of GBLUP for the MY trait (decreased by 9.36%). In terms of RMSE and bias, the WMKRR_pred achieved the lowest RMSE values across all traits, and its biases were similar to or lower than those of GBLUP and GTBLUP_pred [see Additional file 1: Table S3].

After feature selection on genotypes, the predictive ability of WMKRR remained higher than that of GBLUP and GTBLUP for all three traits. Specifically, the predictive ability of WMKRR_pred_FS improved by An average of 5.66% And 6.40% over GBLUP_FS and GTBLUP_pred_FS, ranging from 4.72 to 6.71% And 2.07 to 14.12%, respectively. However, compared to the scenario in which no feature selection was conducted, the predictive abilities of the GBLUP, GTBLUP, and WMKRR methods were not improved after feature selection. Concerning RMSE and bias, WMKRR_pred_FS produced the lowest RMSE values in all scenarios, and no obvious differences in bias were observed between the different methods in the most scenarios, as shown in Table S3 [see Additional file 1: Table S3].

#### Genomic predictive ability in forward validation scenario

**Fig. 7:**
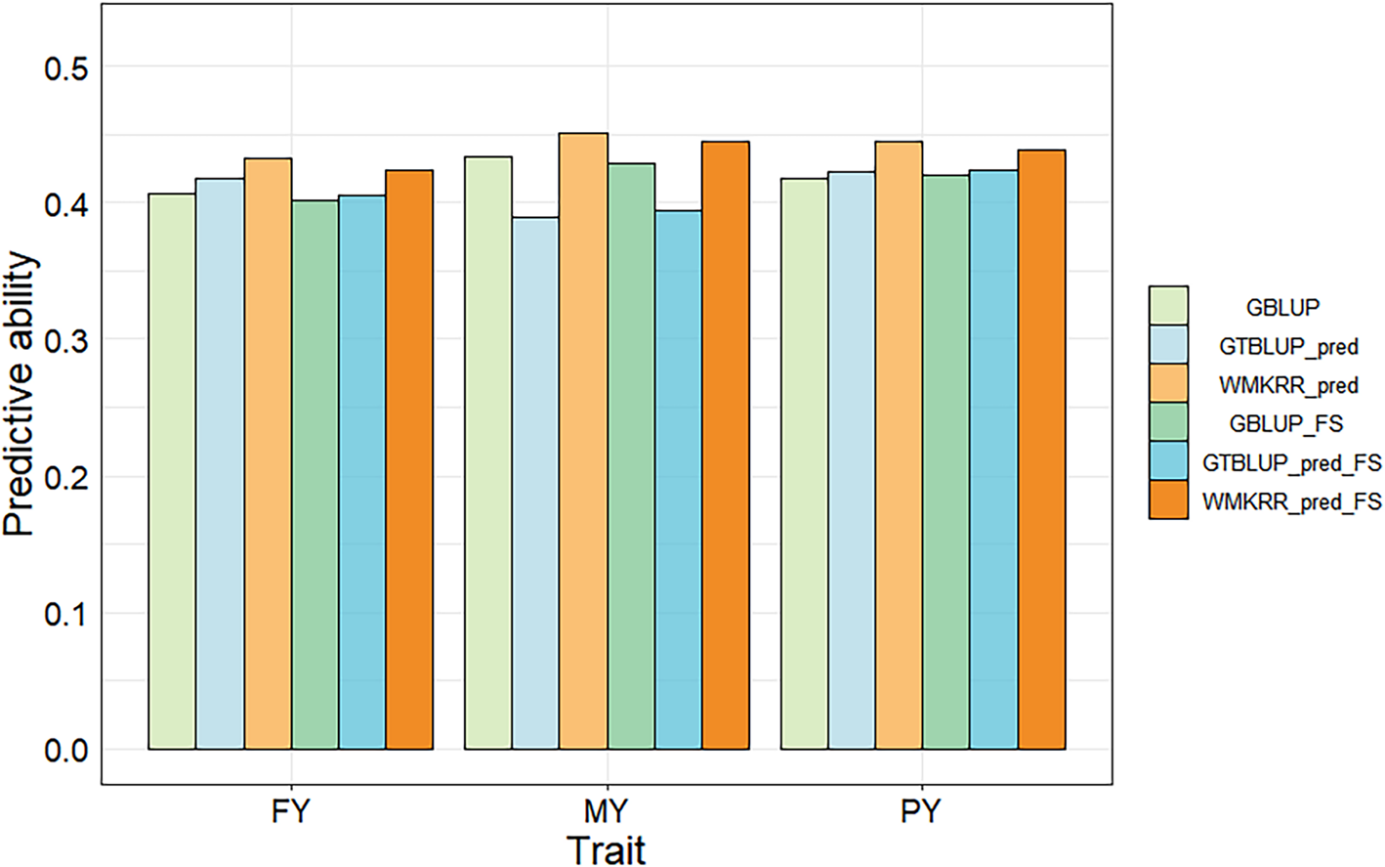
Predictive ability of genomic prediction on the real dairy cattle data in forward validation. FY: fat yield; MY: milk yield; PY: protein yield. GTBLUP_pred: the GTBLUP method based on genotype and predicted gene expression data; WMKRR_pred: the WMKRR method based on genotype and predicted gene expression data; GBLUP_FS: GBLUP with feature selection; GTBLUP_pred_FS: GTBLUP_pred with feature selection; WMKRR_pred_FS: WMKRR_pred with feature selection

Figure 7 shows the genomic predictive abilities on the real dairy cattle data in the forward validation scenario. WMKRR_pred still demonstrated higher predictive ability than GBLUP and GTBLUP_pred. In the absence of feature selection, WMKRR_pred improved by An average of 5.68% And 8.41% over traditional GBLUP and GTBLUP_pred across all traits, with ranges from 3.92 to 6.65% And 3.84 to 15.94%, respectively. In addition, GTBLUP_pred outperformed GBLUP for FY and PY traits (improved by 2.71% And 0.96%, respectively), but performed worse than GBLUP for the MY trait (decreased by 10.37%). The discrepancies for PY and MY compared to cross-validation results may arise from different population partitioning schemes. Forward validation divides the population by age, and its accuracy is often affected by factors such as population size and birth year distribution, while cross-validation may obtain relatively stable results through multiple divisions and predictions. Additionally, WMKRR_pred achieved the lowest RMSE values for all traits [see Additional File 1: Table S4]. After feature selection, WMKRR still demonstrated higher predictive ability. WMKRR_pred_FS improved by An average of 4.66% And 7.06% over GBLUP_FS and GTBLUP_pred_FS, with ranges from 3.73 to 5.74% And 3.54 to 12.94%, respectively. Additionally, WMKRR_pred_FS also achieved the lowest RMSE values for all three traits, as shown in Table S4 [see Additional File 1: Table S4].

## Discussion

Integrative analysis based on multiple omics data is a promising approach that enables leveraging additional information to improve genomic prediction accuracy [20]. In the context of breeding programs, the high cost of collecting omics data currently makes it unlikely to obtain such data for all individuals in a large reference population. However, the decreasing cost of genotyping has made it feasible to acquire large-scale genotypic data. Consequently, predicting omics data based on genotypes and integrating genetically predicted omics data has become a viable approach. In the present work, we extended the KRR algorithm to the weighted multiple kernel form (i.e., WMKRR) that enables simultaneous integration of genomic and predicted transcriptomic information and achieves automatic optimization of kernel weights to improve genomic prediction. Overall, WMKRR demonstrates advantages over conventional genomic prediction approaches, and its advantages are primarily reflected in three aspects:

### Integrating omics data through kernel fusion to reduce overfitting

In recent years, kernel-based data fusion also called kernel fusion [48] has drawn increasing attention. Both data fusion and MKL are facilitated by the excellent closure property in kernel algebra: the sum or weighted sum of kernels is another valid kernel [20]. By utilizing kernel trick, kernel fusion methods can transform kernel matrices generated from different omics data into a unified global kernel with a shared feature space. These methods, along with other machine learning approaches, provide new tools for integrating multi-omics data. In this study, GTBLUP did not demonstrate an improvement in predictive ability in many scenarios. This finding agrees with the previous study of Li et al. [25], in which GTBLUP slightly decreased the combined predictive ability for most traits when integrating transcriptomic data into genomic predictions for nine traits in Drosophila. Xu et al. [49] also observed an analogous result that combining transcriptomic and metabolic data into genomic prediction for six yield-related traits in maize resulted in reduced predictive ability. For GTBLUP, although modelling transcriptomic information as an additional random effect within the genomic prediction model may help capture an extra portion of variance not explained by SNP genotype data alone, the addition of this extra layer in the model is likely to contain overlapping information, increasing collinearity among predictors and potentially leading to overfitting [50]. In contrast, the ridge regression approach in the WMKRR method inherently handles multicollinearity among features effectively [51]. More importantly, WMKRR utilizes the kernel fusion to map different omics data into their respective feature spaces to construct corresponding kernel functions, combining multiple kernels into a composite kernel with weighted contributions and automatically optimizing the weights of each kernel. This approach effectively reduces information redundancy without increasing model complexity, thus mitigating the risk of overfitting.

For ML methods, overfitting is likely to occur when the number of features greatly exceeds the sample size, and feature selection can effectively reduce the risk of overfitting by significantly reducing the number of features [52]. Therefore, in the present study, feature selection for WMKRR demonstrated effectiveness in the WGS-based CattleGTEx data, while no improvement was observed in the real dairy cattle data based on the 50 K SNP panel. Additionally, another possible reason is that univariate feature selection evaluates each feature independently by calculating its statistics with the target variable and selects significantly correlated features, which does not account for interactions or nonlinear relationships among features. Therefore, univariate feature selection is effective on the CattleGTEx data where there is a clear linear relationship between genotypes and simulated phenotypes, but does not show an advantage on the real dairy cattle data with complex traits. Overall, the applications of kernel fusion in genomic prediction are currently limited, but are expected to increase as large-scale omics datasets become more widely available.

### Automatic optimization of kernel weights driven by data

In the present study, the weights of the kernels reflect the contribution of data sources to the genomic prediction task, which were automatically determined through a data-driven Bayesian optimization tuning strategy [36]. This strategy enables WMKRR to autonomously find the optimal proportion of contributions from various omics data to the prediction task, without being influenced by prior information. As the diversity of omics data increases, WMKRR can be easily extended to incorporate additional kernels. This advantage of automatic weight optimization may become more pronounced, since not all data contribute valuable information to prediction and overlapping information may exist among data sources, and WMKRR can naturally reduce their corresponding weights to reduce redundancy. As an emerging hyperparameter optimization strategy, Bayesian optimization algorithm combines Gaussian processes with acquisition functions to reduce the number of evaluations required for the objective function, enabling efficient hyperparameter optimization. Compared to traditional grid search and manual tuning, Bayesian optimization often demonstrated superior prediction performance and much higher tuning efficiency [53, 54]. In this study, aside from kernel weights, other hyperparameters (e.g., regularization constant $\uplambda$ and the Gaussian kernel parameter $\theta$) were also determined through Bayesian optimization algorithm, free from human experience, allowing WMKRR to flexibly adapt to different input patterns of the data and select the hyperparameter values optimal for model fitting.

### Capture of nonlinear structures through Gaussian kernel function

In GBLUP or GTBLUP methods, the genomic relationship matrix G can be viewed as a parametric kernel that only captures genetic values based on additive genetic relationships among individuals. Conversely, the Gaussian kernel used in WMKRR is a non-parametric kernel that can pick up genetic signals regardless of the underlying genetic architecture [25], and studies showed that the Gaussian kernel is capable of capturing higher-order epistasis [55–57]. In this study, the improvement achieved by WMKRR using a linear kernel based on the CattleGTEx data was lower than that achieved using a Gaussian kernel based on the real dairy cattle data, as shown in Figs. 4 and 6. The Gaussian kernel may contribute to the outperformance of WMKRR in the real dairy cattle data. However, it should be noted that the optimization of hyperparameter (e.g. bandwidth parameter $\theta$) plays a crucial role in the Gaussian kernel, maximizing the benefits of kernel methods [53]. This is essential to ensure that kernel methods are optimally utilized not only in the context of genomic prediction but also across all types of prediction problems.

In practical breeding, transcriptome prediction can significantly reduce the need for omics sequencing and associated costs. WMKRR integrates genomes and genetically predicted transcriptomes into a single model, allowing learning the genetic characteristics of different data types. However, it is challenging to accurately predict the expression of all genes because only parts of transcripts exhibit relatively high predictability, and these genetically predictable genes are mostly *cis*-eQTL genes, as the variation of *trans*-eQTL genes within a population is not only determined by their own cis-regulatory elements but also affected by other genes [30]. Hu et al. [30] reported that the genomic prediction accuracy was significantly improved by using predicted values of genetically predictable genes compared to using predicted values from all genes. Therefore, the transcriptome data used in this study includes only genetically predictable genes, rather than all genes across the genome. Notably, for the muscle, liver and blood tissues in the CattleGTEx data (Fig. 3a–c) and the blood tissue in the real dairy cattle data (Fig. 5), there was a high correlation between gene expression prediction accuracy and cis-heritability of gene expression, implicating that the extent to which a gene's expression can be accurately predicted by genetic variants largely depends on its cis-heritability [30]. However, the lower correlation of mammary tissue (Fig. 3d) may be due to the high non-expression rate (i.e., expression level of 0) in the raw gene expression data for this tissue (as shown in Figure S2d [see Additional file 2: Figure S2d], where ~ 98% of genes have a non-expression rate greater than 80%), unlike other tissues, which exhibit much lower non-expression rates in their raw gene expression data [see Additional file 2: Figure S2a–c and Figure S3].

The limited improvement of the WMKRR method on the CattleGTEx dataset may be due to the following reasons: (I) The optimal hyperparameters for ML algorithms are often population-specific [23]. Since the CattleGTEx dataset includes multiple breeds with different genetic backgrounds, and the genetic regulatory mechanisms underlying gene expression may also differ across breeds, different breeds may have distinct optimal hyperparameters. A unified tuning strategy greatly increased the search space for hyperparameters and their instability (results not shown), thereby limiting the advantages of WMKRR. Therefore, stratified modelling based on breed (or population) and the development of breed(population)-specific hyperparameter optimization strategies may be required in multi-breed (population) genomic prediction. (II) In the CattleGTEx data, genomic prediction and gene expression prediction share the same SNPs, which may result in a degree of information redundancy. Weight control and optimization may not fully eliminate this redundancy; further optimization might require assigning different weights to different SNPs (or genes) based on biological information to distinguish their respective contributions [58]. In addition, there remain several potential expansions to the WMKRR framework. (I) Partition the data and construct multiple kernels before kernel fusion. In the application of genomic prediction, the types of omics data available are often limited. However, we can divide each data type into numerous basic kernels, such as feature sets grouped by biological function (e.g., pathway information and interaction networks) or constructed via statistical scoring. This approach may significantly enhance the advantages of MKL and capture more information. (II) Employ nonlinear methods to combine different kernels. As the number of kernels increases, kernels can be combined through products or more complex functional forms to capture interactions among them, providing richer data representations beyond simple linear weighting. (III) Combine WMKRR with ensemble learning. Given the complex data structures and diverse data types in omics (e.g. data generated from different platforms), it is challenging to predefine an optimal kernel function for specific applications [20]. Hence, future efforts should also focus on integrating WMKRR with ensemble learning techniques to enhance scalability with respect to data sources and heterogeneity. Finally, as the quality and quantity of biological annotations improve, understanding how to more effectively utilize prior knowledge to design kernel matrices will gain growing importance for enhancing the predictive performance of models.

## Conclusions

This study extended KRR algorithm to a weighted multiple kernel format (i.e., WMKRR) to integrate genome and genetically predicted transcriptomic information for improving genomic prediction. Our results demonstrated that WMKRR showed higher predictive abilities than traditional GBLUP and GTBLUP methods in both CattleGTEx data and real dairy cattle data. Therefore, although genetically predicted gene expression may not fully represent actual gene expression, integrating genetically predicted gene expression into the WMKRR model enhanced the performance of genomic prediction without increased cost for transcriptome sequencing. This study showed the potential of enhanced genomic breeding application using omics data with no further omics sequencing cost.

## Supplementary Information

**Additional file 1: **Table S1. The pedigree relationship between the 157 cows with transcriptome data and the 5358 cattle without transcriptome data. Table S2 Predictive ability, unbiasedness, and root mean squared error (RMSE) of genomic prediction based on the CattleGTEx data. Table S3 Predictive ability, unbiasedness, and root mean squared error (RMSE) of genomic prediction on three traits of the real dairy cattle data in cross-validation. Table S4 Predictive ability, unbiasedness, and root mean squared error (RMSE) of genomic prediction on three traits of the real dairy cattle data in forward validation

**Additional file 2: **Figure S1. Title: Average imputation accuracy for SNPs with different chromosomes (a) and minor allele frequency (MAF) intervals (b). Figure S2 Distribution of gene non-expression rates in the CattleGTEx data. Figure S3 Distribution of gene non-expression rates in the 157 real dairy cows with transcriptomic data

## Data Availability

The WMKRR software is available on GitHub at https://github.com/Wangxuer521/WMKRR/tree/master. The code for phenotype simulation is available on GitHub at https://github.com/Wangxuer521/Phe_simulation_code/tree/master. The datasets used during the present study are available from the corresponding author on reasonable request.
