# Supplementary material for: Integrating gene expression data via weighted multiple kernel ridge regression improved accuracy of genomic prediction
Source: Genet Sel Evol. 2025 Sep 25;57:48. doi: 10.1186/s12711-025-00997-9 (PMC12465700; doi:10.1186/s12711-025-00997-9)
Supplement: Supplementary file 1 — Additional file 1: Table S1. The pedigree relationship between the 157 cows with transcriptome data and the 5358 cattle without transcriptome data. Table S2 Predictive ability, unbiasedness, and root mean squared error (RMSE) of genomic prediction based on the CattleGTEx data. Table S3 Predictive ability, unbiasedness, and root mean squared error (RMSE) of genomic prediction on three traits of the real dairy cattle data in cross-validation. Table S4 Predictive ability, unbiasedness, and root mean squared error (RMSE) of genomic prediction on three traits of the real dairy cattle data in forward validation [file 12711_2025_997_MOESM1_ESM.docx]

**Table S1. The pedigree relationship between the 157 cows with transcriptome data and the 5358 cattle without transcriptome data**

| **Relationship type** | **Number of individuals in 157 cows** | **Relationship with 5358 cattle** |
| --- | --- | --- |
| Mothers of 5358 cattle | 16 | Mother-offspring relationship |
| Daughters of 5358 cattle | 11 | Mother-daughter relationship |
| Half-siblings of 5358 cattle | 147 | Half-sibling relationship |

**Table S2.** Predictive ability, unbiasedness, and root mean squared error (RMSE) of genomic prediction based on the CattleGTEx data

| Tissue | Method^1^ | Measurement^2^ | | |
| --- | --- | --- | --- | --- |
|  |  | Predictive ability ± SE | Unbiasedness ± SE | RMSE ± SE |
| Liver | GBLUP | 0.407 ± 0.032 | 1.051 ± 0.098 | 1.054 ± 0.030 |
|  | GTBLUP_pred | 0.405 ± 0.032 | 1.037 ± 0.099 | 1.055 ± 0.030 |
|  | GTBLUP_raw | 0.407 ± 0.032 | 1.047 ± 0.099 | 1.054 ± 0.030 |
|  | WMKRR_pred | 0.412 ± 0.031 | 0.936 ± 0.081 | 1.060 ± 0.034 |
|  | WMKRR_raw | 0.415 ± 0.032 | 0.989 ± 0.085 | 1.053 ± 0.030 |
|  | GBLUP_FS | 0.406 ± 0.032 | 0.987 ±0.084 | 1.064 ± 0.034 |
|  | GTBLUP_pred_FS | 0.406 ± 0.032 | 0.987 ±0.084 | 1.054 ±0.030 |
|  | GTBLUP_raw_FS | 0.406 ± 0.032 | 0.987 ±0.084 | 1.054 ±0.030 |
|  | WMKRR_pred_FS | 0.428 ± 0.032 | 0.959 ± 0.081 | 1.045 ± 0.030 |
|  | WMKRR_raw_FS | **0.431** ± 0.031 | **1.008** ± 0.084 | **1.041** ± 0.030 |
| Muscle | GBLUP | 0.530 ± 0.024 | **0.997** ± 0.063 | 1.086 ± 0.027 |
|  | GTBLUP_pred | 0.530 ± 0.024 | 0.994 ± 0.063 | 1.086 ± 0.027 |
|  | GTBLUP_raw | 0.531 ± 0.024 | 0.993 ± 0.063 | 1.086 ± 0.027 |
|  | WMKRR_pred | 0.532 ± 0.024 | 0.942 ± 0.057 | 1.089 ± 0.027 |
|  | WMKRR_raw | 0.536 ± 0.024 | 1.025 ± 0.063 | 1.084 ± 0.027 |
|  | GBLUP_FS | 0.526 ± 0.025 | 0.993 ± 0.063 | 1.092 ± 0.027 |
|  | GTBLUP_pred_FS | 0.527 ± 0.025 | 0.993 ± 0.064 | 1.091 ± 0.027 |
|  | GTBLUP_raw_FS | 0.526 ± 0.025 | 0.993 ± 0.064 | 1.091 ± 0.027 |
|  | WMKRR_pred_FS | 0.542 ± 0.024 | 0.967 ± 0.057 | 1.080 ± 0.027 |
|  | WMKRR_raw_FS | **0.544** ± 0.024 | 1.009 ± 0.060 | **1.079** ± 0.027 |
| Blood | GBLUP | 0.516 ± 0.025 | **1.001** ± 0.060 | 1.023 ± 0.026 |
|  | GTBLUP_pred | 0.516 ± 0.025 | **0.999** ± 0.060 | 1.023 ± 0.026 |
|  | GTBLUP_raw | 0.517 ± 0.025 | **0.999** ± 0.060 | 1.026 ± 0.028 |
|  | WMKRR_pred | 0.517 ± 0.025 | 0.918 ± 0.054 | 1.024 ± 0.026 |
|  | WMKRR_raw | 0.520 ± 0.025 | 1.008 ± 0.059 | 1.022 ± 0.026 |
|  | GBLUP_FS | 0.514 ± 0.025 | 0.972 ± 0.058 | 1.029 ±0.029 |
|  | GTBLUP_pred_FS | 0.514 ± 0.025 | 0.972 ± 0.058 | 1.027 ±0.026 |
|  | GTBLUP_raw_FS | 0.515 ± 0.025 | 0.972 ± 0.058 | 1.027 ±0.026 |
|  | WMKRR_pred_FS | 0.522 ± 0.025 | 0.954 ± 0.056 | 1.022 ± 0.026 |
|  | WMKRR_raw_FS | **0.524** ± 0.025 | 1.010 ± 0.059 | **1.020** ± 0.026 |
| Mammary | GBLUP | 0.671 ± 0.021 | 1.004 ± 0.046 | 0.876 ± 0.028 |
|  | GTBLUP_pred | 0.666 ± 0.022 | 0.965 ± 0.047 | 0.881 ± 0.029 |
|  | GTBLUP_raw | 0.668 ± 0.021 | 0.993 ± 0.047 | 0.880 ± 0.029 |
|  | WMKRR_pred | 0.673 ± 0.020 | 1.059 ± 0.048 | 0.881 ± 0.029 |
|  | WMKRR_raw | **0.675** ± 0.021 | 1.011 ± 0.046 | **0.873** ± 0.028 |
|  | GBLUP_FS | 0.669 ± 0.022 | 1.032 ± 0.050 | 0.879 ± 0.028 |
|  | GTBLUP_pred_FS | 0.666 ± 0.023 | **1.003** ± 0.050 | 0.882 ± 0.029 |
|  | GTBLUP_raw_FS | 0.665 ± 0.022 | **1.003** ± 0.050 | 0.883 ± 0.029 |
|  | WMKRR_pred_FS | 0.673 ± 0.022 | 1.017 ± 0.048 | 0.875 ± 0.029 |
|  | WMKRR_raw_FS | 0.674 ± 0.021 | 1.023 ± 0.046 | 0.875 ± 0.029 |

^1^ GTBLUP_pred/raw: the GTBLUP method based on genotype and predicted/actual gene expression data; WMKRR_pred/raw: the WMKRR method based on genotype and predicted/actual gene expression data; GBLUP_FS: GBLUP with feature selection; GTBLUP_pred/raw_FS: GTBLUP_pred/raw with feature selection; WMKRR_pred/raw_FS: WMKRR_pred/raw with feature selection.

^2^ Predictive ability: the correlation between simulated phenotypes and predicted values of the validation population; Unbiasedness: the slope of the regression of simulated phenotypes onto the predicted values.

Bold fonts in the same column for the same tissue denotes the highest predictive ability, the lowest bias, or the smallest root mean square error (RMSE).

**Table S3.** Predictive ability, unbiasedness, and root mean squared error (RMSE) of genomic prediction on three traits of the real dairy cattle data in cross-validation

| Trait^1^ | Method^2^ | Measurement^3^ | | |
| --- | --- | --- | --- | --- |
|  |  | Predictive ability ± SE | Unbiasedness ± SE | RMSE ± SE |
| FY | GBLUP | 0.424±0.013 | 1.071±0.040 | 42.613±0.351 |
|  | GTBLUP_pred | 0.434±0.012 | 1.042±0.036 | 42.469±0.381 |
|  | WMKRR_pred | **0.446**±0.012 | 1.041±0.033 | **42.317**±0.375 |
|  | GBLUP_FS | 0.424±0.012 | 1.008±0.035 | 42.604±0.352 |
|  | GTBLUP_pred_FS | 0.431±0.012 | 0.983±0.032 | 42.515±0.375 |
|  | WMKRR_pred_FS | 0.444±0.012 | **0.997**±0.032 | 42.335±0.376 |
| MY | GBLUP | 0.470±0.013 | 1.158±0.039 | 1125.010±13.077 |
|  | GTBLUP_pred | 0.426±0.015 | 1.517±0.082 | 1142.564±13.476 |
|  | WMKRR_pred | **0.497**±0.013 | 1.126±0.034 | **1112.551**±12.509 |
|  | GBLUP_FS | 0.462±0.014 | 1.085±0.038 | 1126.968±13.045 |
|  | GTBLUP_pred_FS | 0.432±0.016 | 1.407±0.072 | 1137.223±13.323 |
|  | WMKRR_pred_FS | 0.493±0.013 | **0.985**±0.030 | 1112.867±12.649 |
| PY | GBLUP | 0.469±0.014 | 1.108±0.036 | 35.609±0.359 |
|  | GTBLUP_pred | 0.485±0.012 | 1.018±0.029 | 35.367±0.361 |
|  | WMKRR_pred | **0.496**±0.012 | 1.091±0.029 | **35.240**±0.350 |
|  | GBLUP_FS | 0.467±0.014 | 1.070±0.034 | 35.619±0.354 |
|  | GTBLUP_pred_FS | 0.483±0.013 | **0.986**±0.029 | 35.395±0.354 |
|  | WMKRR_pred_FS | 0.493±0.012 | 1.199±0.032 | 35.383±0.353 |

^1^ FY: fat yield; MY: milk yield; PY: protein yield.

^2^ GTBLUP_pred: the GTBLUP method based on genotype and predicted gene expression data; WMKRR_pred: the WMKRR method based on genotype and predicted gene expression data; GBLUP_FS: GBLUP with feature selection; GTBLUP_pred_FS: GTBLUP_pred with feature selection; WMKRR_pred_FS: WMKRR_pred with feature selection.

^3^ Predictive ability: the correlation between DRP and predicted values of the validation population divided by the mean accuracy of DRP in validation data; Unbiasedness: the slope of the regression of DRP onto the predicted values

Bold fonts in the same column for the same trait denotes the highest predictive ability, the lowest bias, or the smallest root mean square error (RMSE).

**Table S4.** Predictive ability, unbiasedness, and root mean squared error (RMSE) of genomic prediction on three traits of the real dairy cattle data in forward validation

| Trait^1^ | Method^2^ | Measurement^3^ | | |
| --- | --- | --- | --- | --- |
|  |  | Predictive ability | Unbiasedness | RMSE |
| FY | GBLUP | 0.406 | 1.104 | 45.585 |
|  | GTBLUP_pred | 0.417 | 1.123 | 45.448 |
|  | WMKRR_pred | **0.433** | 1.171 | **45.254** |
|  | GBLUP_FS | 0.401 | **1.063** | 45.645 |
|  | GTBLUP_pred_FS | 0.405 | 1.121 | 45.592 |
|  | WMKRR_pred_FS | 0.424 | 1.155 | 45.381 |
| MY | GBLUP | 0.434 | 1.183 | 1251.074 |
|  | GTBLUP_pred | 0.389 | 1.516 | 1273.253 |
|  | WMKRR_pred | **0.451** | **0.997** | **1239.667** |
|  | GBLUP_FS | 0.429 | 1.044 | 1250.946 |
|  | GTBLUP_pred_FS | 0.394 | 1.556 | 1271.795 |
|  | WMKRR_pred_FS | 0.445 | 1.055 | 1243.228 |
| PY | GBLUP | 0.418 | **0.998** | 39.048 |
|  | GTBLUP_pred | 0.422 | 1.003 | 39.007 |
|  | WMKRR_pred | **0.445** | 1.199 | **38.804** |
|  | GBLUP_FS | 0.420 | 1.041 | 39.046 |
|  | GTBLUP_pred_FS | 0.424 | 1.080 | 38.947 |
|  | WMKRR_pred_FS | 0.439 | 1.022 | 38.841 |

^1^ FY: fat yield; MY: milk yield; PY: protein yield.

^2^ GTBLUP_pred: the GTBLUP method based on genotype and predicted gene expression data; WMKRR_pred: the WMKRR method based on genotype and predicted gene expression data; GBLUP_FS: GBLUP with feature selection; GTBLUP_pred_FS: GTBLUP_pred with feature selection; WMKRR_pred_FS: WMKRR_pred with feature selection.

^3^ Predictive ability: the correlation between DRP and predicted values of the validation population divided by the mean accuracy of DRP in validation data; Unbiasedness: the slope of the regression of DRP onto the predicted values

Bold fonts in the same column for the same trait denotes the highest predictive ability, the lowest bias, or the smallest root mean square error (RMSE).
