# Supplementary material for: Integrating gene expression data via weighted multiple kernel ridge regression improved accuracy of genomic prediction
Source: Genet Sel Evol. 2025 Sep 25;57:48. doi: 10.1186/s12711-025-00997-9 (PMC12465700; doi:10.1186/s12711-025-00997-9)
Supplement: Supplementary file 2 — Additional file 2: Figure S1. Title: Average imputation accuracy for SNPs with different chromosomes (a) and minor allele frequency (MAF) intervals (b). Figure S2 Distribution of gene non-expression rates in the CattleGTEx data. Figure S3 Distribution of gene non-expression rates in the 157 real dairy cows with transcriptomic data [file 12711_2025_997_MOESM2_ESM.docx]

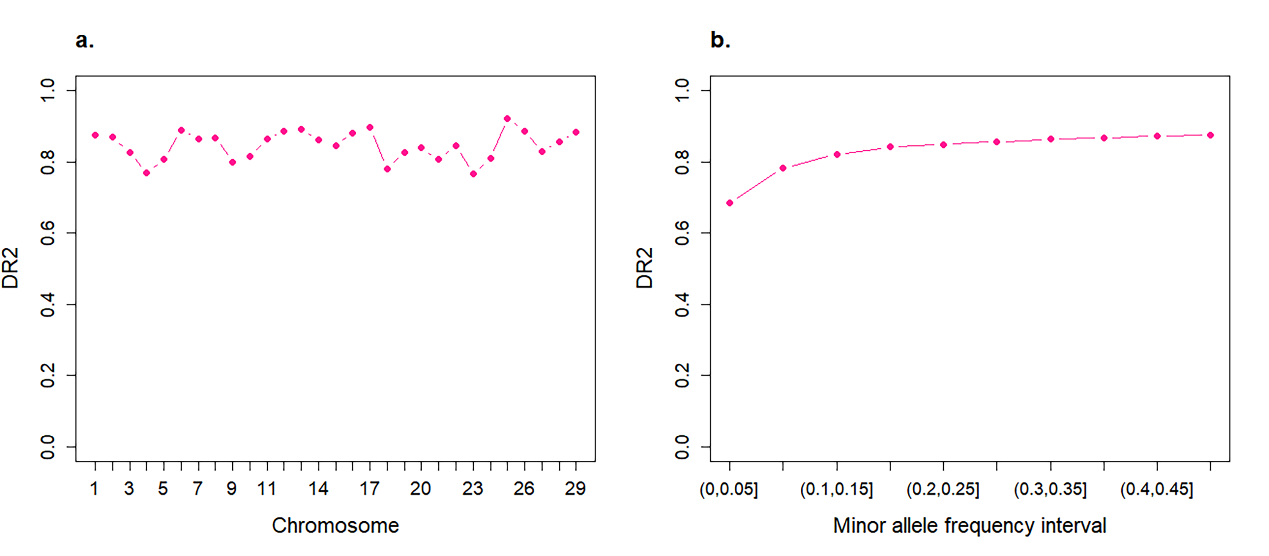


**Figure S1.** Average imputation accuracy for SNPs with different chromosomes (a) and minor allele frequency (MAF) intervals (b).

DR2: average estimated squared correlation between the imputed and the true allele dose.


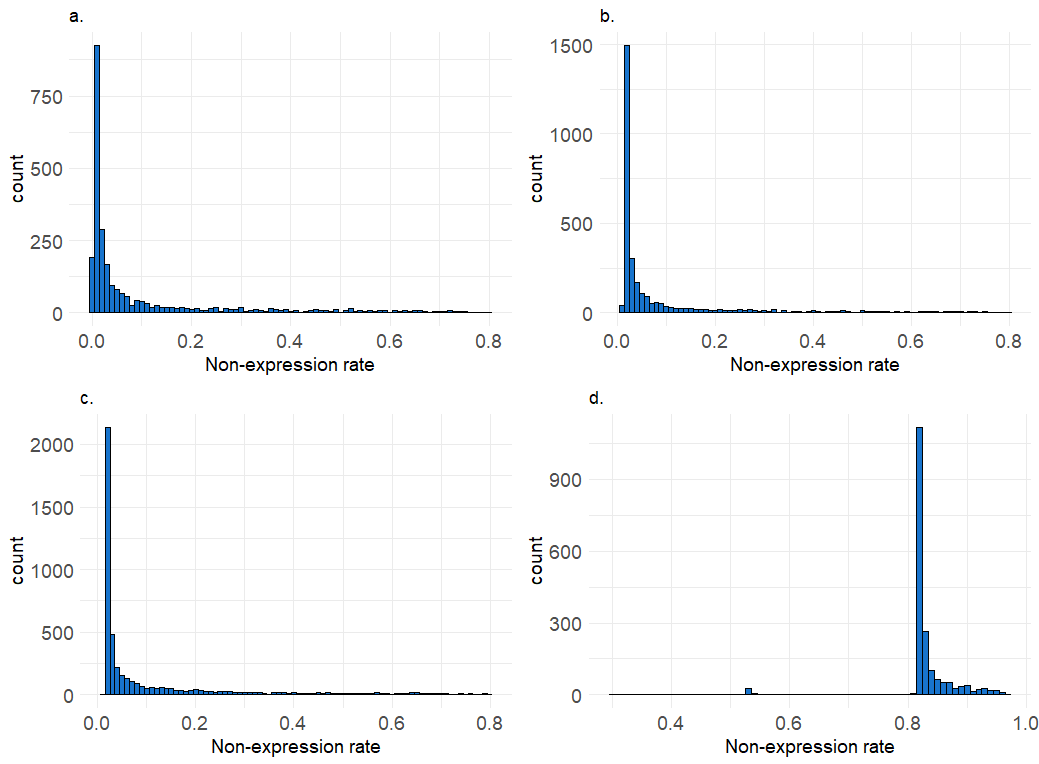


**Figure S2.** Distribution of gene non-expression rates in the CattleGTEx data

a. Distribution of gene non-expression rates in muscle; b. Distribution of gene non-expression rates in liver; c. Distribution of gene non-expression rates in blood; d. Distribution of gene non-expression rates in mammary.


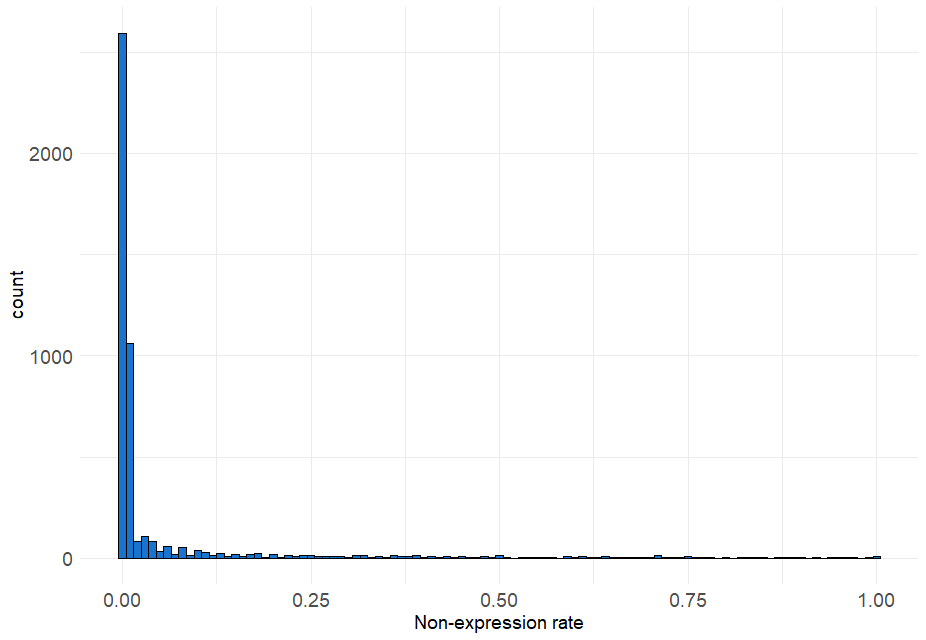


**Figure S3.** Distribution of gene non-expression rates in the 157 real dairy cows with transcriptomic data
